# Influences of drying temperature and storage conditions for preserving the quality of maize postharvest on laboratory and field scales

**DOI:** 10.1038/s41598-020-78914-x

**Published:** 2020-12-15

**Authors:** Paulo Carteri Coradi, Vanessa Maldaner, Éverton Lutz, Paulo Vinícius da Silva Daí, Paulo Eduardo Teodoro

**Affiliations:** 1 Campus Cachoeira Do Sul, Federal University of Santa Maria, Ernesto Barros Street, 1345 - Santo Antônio, Cachoeira Do Sul, RS 96506-322 Brazil; 2 Department of Agricultural Engineering, Federal University of Santa Maria, Avenue Roraima, 1000, Camobi, Santa Maria, RS 97105900 Brazil; 3 Campus de Chapadão Do Sul, Federal University of Mato Grosso Do Sul, Chapadão Do Sul, Mato Grosso Do Sul, MS 79560-000 Brazil; 4 Present Address: Campus Cachoeira Do Sul, Federal University of Santa Maria, Ernesto Barros Street, 1345 - Santo Antônio, Cachoeira Do Sul, RS 96506-322 Brazil

**Keywords:** Biotechnology, Engineering

## Abstract

Drying and storage methods are fundamental for maintaining the grain quality until processing. Therefore, the aim of this study was to evaluate the associations of the drying temperature with storage systems and conditions as a strategy for preserving the quality of maize grain postharvest on laboratory and field scales. An increase in temperature accelerated the reduction in grain moisture, but increased the deterioration. The wetting during the storage period reduced the grain quality. Hermetic and aerated storage systems maintained the chemical quality of the grains. The control with healthy and whole corn dried at 80 °C and stored in silos with natural aeration provided a satisfactory quality, equivalent to those of controlled drying and storage under airtight conditions and at low temperatures. Different conditions of drying and storage of corn on the laboratory and field scales were evaluated, which provides an appropriate management of these operations to maintain the grain quality.

## Introduction

Corn is produced and consumed on a large scale owing to its nutritional value and different forms of use in the food and biofueyl industries^1–3^. After the harvest, the corn is sold or stored for better market prices. Quantitative and qualitative losses with very variable magnitudes occur at the harvest and in all postharvest stages, transport, handling, drying, storage, processing, marketing, and endpoint distribution to consumers^4,5^.

The postharvest losses of grains are in the range of 25–30% of the produced value^6–8^. Timely drying aims to reduce the water content for safe storage. Depending on the type of drying, it may compromise the physicochemical quality of the grains and increase the risks of quantitative and qualitative losses in the storage stage^9–12^. Thus, for a proper management, the drying air and grain mass temperatures, initial and final grain moisture contents, air and grain flow in the dryer, and ambient air conditions must be monitored^13–15^. The control of these parameters reduces the chemical and physical damages to the grains during the simultaneous heat and mass transfer. Modeling of this process, seeking balance and efficiency, between the drying speed and batch capacity, is required for different grains and grain genotypes^16–21^.

The main factors that affect the quality of grains during storage are the temperature and water content, which are related to the product respiration and presence of microorganisms^22–24^. However, higher temperature and water content of the stored grain mass lead to a higher biological activity of the grain and consequently to a faster deterioration^25–27^. Excessive drying of the grains, reducing the water content below the ideal storage values, may lead to quantitative and qualitative losses of the grain mass and losses to the storekeeper at the time of commercialization^28–30^.

However, the application of the drying technique to correct the migration of moisture in the grains during storage, with either natural air or heated air, may also lead to changes in the physical quality and consequently nutritional quality of the grains^31,32^. Aeration aims to cool the mass of grains stored in the silos and may be operated continuously or intermittently until the mass of the grains reaches the desired temperature levels. The reduction in the temperature of the grains decreases the speed of the biochemical and metabolic reactions, while maintaining the initial grain quality characteristics. Some studies using artificial cooling have been carried out on beans, soybeans, and rice, while only few studies have been carried out with corn kernels^33,34^.

Technologies that reduce the concentration of oxygen and increase the content of CO_2_ in the storage environment may reduce the respiration of the grains, making the environment unsuitable for the development of microorganisms and insect pests^35,36^. Therefore, choosing the best drying and storage alternatives and monitoring system according to the region can minimize the losses. Therefore, this study was carried out to understand the drying kinetics and effects on the quality of corn kernels at high temperatures associated with the storage with different technologies. The aim was to evaluate the association of drying temperatures with the technology and storage conditions as a strategy for preserving the quality of maize grain postharvest on laboratory and field scales.

## Results and discussion

### Quality of corn grains subjected to drying and storage under different production-scale conditions

**Figure 1:**
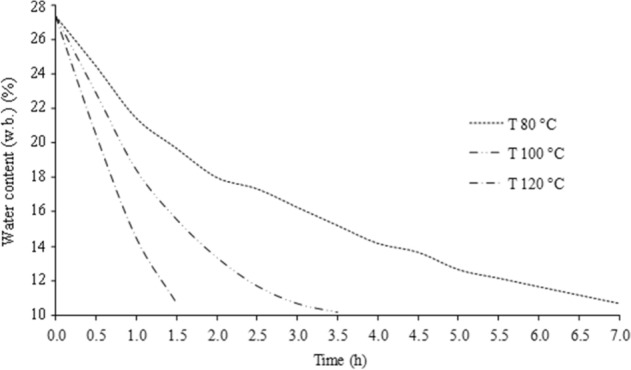
Drying curves of corn kernels at 80, 100, and 120 °C and airflow of 220 m^3^ h^−1^.

Figure 1 shows the drying curves of corn, which describe the time required to reach the desired storage water content (< 12%). The main differential of the process was the increase in the drying air temperature.

The physical properties of corn, owing to the different shapes, storage times, and qualities, had significant differences at a probability of 5%. Table 1 show that the storage of the corn with the application of the aeration system and airtight system preserved the physical dimensions of the corn. The largest physical changes occurred in grains stored without aeration and in bags. Regardless of the used system, the storage time influenced the physical dimensions. Table 2 shows the mass of whole corn with preserved physical characteristics, regardless of the shape and storage. Conservation of the physical and mechanical properties is important for storage, design, construction, and operation of various equipment used in postharvest operations^37–43^.

**Table 1:** Physical dimensions of the corn kernels stored under different systems and durations.

Storage	Whole corn			Cracked corn			Normal corn		
	Time (months)			Time (months)			Time (months)		
	Zero	Three	Six	Zero	Three	Six	Zero	Three	Six
**Length** (mm)*									
Aerated	11.89 aC	10.70 aA	10.98 aB	9.15 aB	08.01 aA	09.17 aB	11.53 aA	10.56 aA	10.93 aB
Airtight	11.89 aC	11.51 bB	11.25 bA	9.15 aA	10.54 cC	09.61 bB	11.53 aB	11.33 bA	11.30 bA
Bag	11.89 aB	11.46 bA	11.52 bA	9.15 aA	09.15 bA	12.23 dB	11.53 aB	11.40 bB	11.43 cB
Non-aerated	11.89 aC	11.51 bB	11.38 bA	9.15 aA	10.31 cC	10.21 cB	11.53 aB	11.35 bA	11.48 cB
**Width** (mm)*									
Aerated	7.98 aB	8.18 cC	7.82 cA	6.94 aC	5.94 aA	6.79 aB	7.94 aB	8.02 bB	7.66 aA
Airtight	7.98 aC	7.75 aB	7.68 aA	6.94 aB	6.87 cA	6.84 aA	7.94 aB	7.79 bA	7.74 bA
Bag	7.98 aB	7.72 aA	7.75 bA	6.94 aC	6.31 bA	6.76 aB	7.94 aB	7.82 aA	7.83 cA
Non-aerated	7.98 aB	7.89 bA	7.84 cA	6.94 aA	7.28 dB	7.03 bA	7.94 aC	7.81 aB	7.72 bA
**Thickness** (mm)*									
Aerated	7.98 aC	4.40 aA	4.60 cB	4.21 aB	4.86 aC	3.83 aA	4.05 aA	4.50 aA	4.49 bB
Airtight	7.98 aB	4.40 aA	4.36 aA	4.58 cA	4.86 aB	4.46 cA	4.90 dD	4.50 aA	4.59 cB
Bag	7.98 aB	4.40 aA	4.43 bA	4.44 bB	4.86 aC	4.19 bA	4.37 bB	4.50 aA	4.39 aA
Non-aerated	7.98 aC	4.40 aA	4.64 cB	4.49 bA	4.86 aB	4.93 dB	4.77 cC	4.50 aA	4.63 dB
**Volume** (mm^3^)*									
Aerated	218 aC	208 bB	188 aA	160 aC	097 aA	132 aB	215 aC	199 aB	187 aA
Airtight	218 aB	202 aA	206 bA	160 aA	169 cB	171 bB	215 aC	210 bB	207 bA
Bag	218 aB	204 aA	206 bA	160 aB	129 bA	190 cC	215 aB	203 aA	206 bA
Non-aerated	218 aB	219 cB	208 bA	160 aA	191 dC	177 bB	215 aB	213 bB	208 bA
**Projected area** (mm^2^)*									
Aerated	40.99 aC	38.13 aB	36.07 aA	34.61 aC	24.22 aA	29.18 aB	40.52 aB	36.95 aA	36.39 aA
Airtight	40.99 aA	39.07 aA	40.24 cA	34.61 aA	36.95 cB	36.97 bB	40.52 aA	40.53 cA	40.20 cA
Bag	40.99 aA	39.72 aA	39.94 bA	34.61 aB	30.04 bA	40.57 cC	40.52 aA	38.97 bA	39.46 bA
Non-aerated	40.99 aA	41.61 bA	39.90 bA	34.61 aA	39.43 dC	37.73 bB	40.52 aA	40.97 cA	40.58 cA
**Sphericity** (%)*									
Aerated	0.63 aA	0.69 bA	0.65 aA	0.74 aB	0.72 bB	0.70 bA	0.65 aA	0.69 cB	0.66 aA
Airtight	0.63 aA	0.64 aA	0.65 aA	0.74 aC	0.65 aA	0.71 bB	0.65 aA	0.66 bA	0.65 aA
Bag	0.63 aA	0.64 aA	0.64 aA	0.74 aC	0.69 bB	0.63 aA	0.65 aA	0.64 cA	0.64 aA
Non-aerated	0.63 aA	0.65 aA	0.65 aA	0.74 aB	0.70 bA	0.69 bA	0.65 aA	0.66 bA	0.65 aA
**Circularity** (%)*									
Aerated	0.79 aC	0.61 bB	0.59 aA	0.72 aC	0.64 bB	0.55 aA	0.85 aB	0.62 bA	0.65 aA
Airtight	0.79 aB	0.69 aA	0.69 bA	0.72 aA	0.70 cA	0.96 dB	0.85 aB	0.67 bA	0.70 bA
Bag	0.79 aC	0.59 cA	0.74 cB	0.72 aC	0.55 aA	0.82 cB	0.85 aC	0.54 aA	0.68 bB
Non-aerated	0.79 aC	0.67 aA	0.73 cB	0.72 aB	0.65 bA	0.75 bB	0.85 aC	0.74 cB	0.68 bA

*Means followed by the capital letter in the line for each storage time and lowercase in the column for each storage form, do not differ at 5% probability.

**Table 2:** Alterations in the physical mass of shelled corn stored under different systems and durations.

Storage	Cracked corn			Normal corn					
	Time (months)			Time (months)			Time (months)		
	Zero	Three	Six	Zero	Three	Six	Zero	Three	Six
**Porosity** (%)*									
Aerated	44.24 aA	61.55 aB	60.45 aB	46.16 aA	65.20 aB	64.03 aB	46.25 aA	64.91 aB	65.15 aB
Airtight	44.24 aA	65.68 bC	64.06 bB	46.16 aA	66.94 bcB	66.38 bB	46.25 aA	66.67 bB	65.47 aB
Bag	44.24 aA	61.55 aC	58.97 aB	46.16 aA	67.96 cC	66.49 bB	46.25 aA	65.82 abB	65.87 aB
Non-aerated	44.24 aA	64.35 bB	63.96 bB	46.16 aA	65.98 abB	67.10 bB	46.25 aA	66.59 bB	66.16 aB
**Bulk density apparent** (kg m^−3^)*									
Aerated	740 aB	730 bA	730 bA	760 aA	770 aB	770 aB	750 aA	780 aB	800 bC
Airtight	740 aA	760 dB	770 dC	760 aA	770 aB	800 cC	750 aA	790 bB	810 cC
Bag	740 aC	690 aA	700 aB	760 aA	780 bB	810 dC	750 aA	790 bB	810 cC
Non-aerated	740 aA	750 cB	750 cC	760 aA	770 aB	790 bC	750 aA	780 aB	790 aC
**Thousand kernel weight** (g)*									
Aerated	220 aC	125 aA	143 aB	291 aC	283 aB	272 aA	298 aA	312 dB	294 aA
Airtight	220 aB	216 cA	220 bB	291 aA	295 bB	289 bA	298 aB	300 cB	289 bA
Bag	220 aC	150 bB	140 aA	291 aA	300 cB	295 bA	298 aA	305 bB	298 cA
Non-aerated	220 aB	234 dA	230 cA	291 aA	301 cB	300 cB	298 aB	299 aB	294 aA

*Means followed by the capital letter in the line for each storage time and lowercase in the column for each storage form, do not differ at 5% probability.

In a specific case of maize, equipment and operations, when properly sized and operated, can rarely generate kernel cracking and consequently reduction in market prices. To minimize the production costs for a higher competitiveness and improve the quality of the final product, determination and knowledge of the behaviors of corn grain properties are the main factors contributing to proper development processes and simulations to improve the production system of the crop^44,45^. Several factors can interfere with the bulk density, porosity, and weight of corn kernels, including those associated with farming, such as the planting time, incidence of sunlight or excessive shading, temperature, planting density, harvest, transport, drying and storing^46,47^, type of hybrid, and physiological maturity.

Table 2 shows that the porosity of the corn increased with the storage time, regardless of the grain conditions. Between the storage systems, no significant differences in porosity were observed. The porosities of the masses of wheat, rice, and corn are usually in the range of 40–45% of the intergranular spaces, according to the results obtained at zero storage time (the effects of the storage time have also been reported^6^). The analysis of the bulk density showed that the increase in storage time reduced the grain mass in all forms of storage. The worst density results were observed in storage silos without an aeration system. The airtight storage best preserved the initial weight of the grains (thousand grain weight) over time, followed by the storage system in sacks and storage silo with an aeration system.

These results are consistent with those for most agricultural grains. The bulk density is an important parameter to consider when receiving grains^48^. Commonly used by the agribusiness, the determination of the apparent density is an evaluation criterion for product quality and helps determine market prices. The apparent density also corresponds to the weight of the grain mass contained in given volume, expressed in kilogram per cubic meter. Information on porosity, bulk density, and thousand kernel weights is considered of importance for studies involving heat and mass transfer and air movement in granular masses. Together with the water content, volume, density, and porosity, these data are basic parameters for the study of drying conditions and storage of agricultural products and consequently facilitate the prediction of loss of quality of the material until the time of marketing.

According to the results (Table 3), significant differences in the percentage of germination and electrical conductivity of the stored maize grains were observed owing to the triple interaction between the type of grain storage system and duration. In general, a decrease in the percentage of germination of corn kernels stored over time was observed, regardless of the storage medium. The worst results were observed for broken corn kernels. However, according to the system comparison, the corn stored hermetically exhibited higher germination percentages over the storage time. Seeds of wheat, oat (*Avena sativa* L.), and maize (*Zea mays* L.) were well stored in airtight glass containers for five years with controlled temperature and relative air humidity^49^. Both wheat and oat exhibited no significant variations in germination percentage, while the corn kernels exhibited a significant decrease after five years of storage.

**Table 3:** Physical quality of corn stored under different systems and durations.

Storage	Cracked corn			Normal corn			Whole corn		
	Time (months)			Time (months)			Time (months)		
	Zero	Three	Six	Zero	Three	Six	Zero	Three	Six
**Electrical conductivity** (µS cm^−1^ g^−1^)*									
Aerated	678 aA	955 cB	961 dB	169 aA	317 dB	429 cC	114 aA	335 dB	329 dB
Airtight	678 aC	511 aB	428 aA	169 aB	161 aB	150 aA	114 aA	148 bC	138 aB
Bag	678 aB	658 bA	781 bC	169 aB	156 aA	167 bB	114 aA	133 aB	145 bC
Non-aerated	678 aC	351 aA	466 cB	169 aA	170 cA	168 bA	114 aA	157 cB	209 cC
**Germination** (%)*									
Aerated	40.50 aC	08.00 aB	05.50 aA	87.50 aC	44.50 aB	21.00 aA	97.50 aB	92.00 aA	93.50 aA
Airtight	40.50 aA	54.00 dC	39.50 cB	87.50 aA	97.50 bC	95.00 bB	97.50 aA	98.50 bA	98.50 bA
Bag	40.50 aB	47.50 cB	25.50 bA	87.50 aA	94.50 aB	94.50 bB	97.50 aA	97.50 bA	96.50 bA
Non-aerated	40.50 aB	37.50 bA	44.50 dB	87.50 aA	95.00 aB	95.00 bB	97.50 aB	96.00 bB	94.00 aA

*Means followed by the capital letter in the line for each storage time and lowercase in the column for each storage form, do not differ at 5% probability.

In any form of storage, the electrical conductivity of the solution increased over time (Table 3). The grains stored with the aeration system were most affected, while the airtight form of grain storage led to lower electrical conductivities, regardless of the corn lot. Considering the types of grains, the differences in electrical conductivities in stored broken grains were notorious, which indicates a faster deterioration of the cell wall membrane. Grains with higher electrical conductivities are characterized by a higher cell membrane degradation and consequently smaller force^50^. The electrical conductivity of the solution containing the seeds can be used to evaluate this effect, as the conductivity is related to the amount of ions leached into the solution, which is directly associated with the cell membrane integrity. Poorly structured damaged cells and membranes are generally associated with seed deterioration and thus low vigor^51^. The lowest values, corresponding to the lowest level of ions, indicate a high physiological vigor and less intense disorganization of the cell membrane system^52^.

Table 4 shows significant changes (*P* < 0.05) in the water content, protein, ash content, and acid value of the corn grains as a function of the grain shape, storage time, and quality of maize. An increase in water content over the storage time was observed, regardless of the corn lot. The storage with aeration and low-quality grains (broken) led to a larger increase in water content, while no difference between the types of grains in the storage without aeration was observed. In the storage in sacks, batch-to-batch consistency of corn prevailed upon increasing the water content, which could be observed in lots of whole grains and broken grains. In the airtight storage, a higher increase in water level in the broken grains was observed.

**Table 4:** Physical–chemical quality of corn kernels stored under different systems and durations.

Storage	Cracked corn		Normal corn		Whole corn	
	Time (months)		Time (months)		Time (months)	
	Zero	Six	Zero	Six	Zero	Six
**Water content (w.b.)** (%)						
Aerated	10.16 Ba	12.55 Ab	9.86 Aa	12.96 Bb	9.85 Aa	12.68 Ab
Airtight	10.19 Bb	12.18 Bb	9.86 Aa	12.08 Bb	9.85 Aa	11.76 Ab
Bag	10.19 Ba	12.70 Bb	9.86 Aa	11.98 Ab	9.85 Aa	12.56 Bb
Non-aerated	10.19 Ba	12.97 Ab	9.86 Aa	12.72 Ab	9.85 Aa	12.83 Ab
Crude protein (%)						
Aerated	9.02 Ab	7.59 Aa	9.08 Ab	8.06 Aa	10.14 Bb	8.44 Ba
Airtight	9.02 Ab	8.89 Ba	9.08 Ab	8.43 Aa	10.14 Bb	8.99 Ba
Bag	9.02 Ab	8.79 Ba	9.08 Ab	8.66 Aa	10.14 Bb	8.77 Bb
Non-aerated	9.02 Ab	8.68 Aa	9.08 Ab	9.10 Ca	10.14 Bb	8.89 Ba
**Acid index (NAOH mL)**						
Aerated	1.94 Ab	1.85 Ca	2.76 Cb	1.50 Aa	2.24 Bb	1.58 Ba
Airtight	1.94 Ab	1.65 Ba	2.76 Cb	1.83 Ca	2.24 Bb	1.57 Aa
Bag	1.94 Ab	1.62 Aa	2.76 Cb	1.85 Ca	2.24 Bb	1.75 Ba
Non-aerated	1.94 Ab	1.77 Ba	2.76 Cb	1.65 Aa	2.24 Bb	1.82 Ca
**Ashes** (%)						
Aerated	1.23 Ab	0.98 Ba	1.26 Bb	1.26 Bb	1.35 Cb	1.25 Cb
Airtight	1.23 Ab	0.67 Aa	1.26 Bb	0.99 Ba	1.35 Cb	0.98 Ba
Bag	1.23 Ab	1.05 Aa	1.26 Bb	1.14 Ba	1.35 Cb	1.09 Aa
Non-aerated	1.23 Cb	0.86 Aa	1.26 Bb	0.84 Aa	1.35 Cb	1.03 Ba

*Means followed by the capital letter in the line for each storage time and lowercase in the column for each storage form, do not differ at 5% probability.

The factor of largest variation in terms of water content was the storage form, although the grains stored in the hermetic system maintained the quality. Among the grain lots, the variations in water content were similar, i.e., the effects of the storage environment prevailing with respect to the quality of the grains were similar between the normal batches and broken grains. The grain storage bins with aeration and airtight systems provided the best results for crude protein. Among the types of grains, the percentage of protein was significantly high for lots with whole grains. However, the storage time had the strongest influence on the reduction in protein percentage for whole corn grains, regardless of the storage form.

The crude protein of broken corn kernels had diference final values in the non-airtight system, possibly because when the analysis of crude protein was performed, the fungal protein was also analyzed. Thus, the specific content is the sum of grain protein and fungal protein^53,54^. These results show that the storage time had a small influence on the storage form in the analysis of the mass of grains broken and mixed differently for whole grains. According to these studies, the crude protein serves as a primary source of carbon and nitrogen for the growth and metabolism of fungi. Fungal growth can occur, even at low levels, in the airtight system for oxygen. In addition, an initial increase in crude protein content of the grain may occur, but to a lesser extent compared to non-hermetic systems. In non-airtight systems, the thermal exchange and moisture are lower than those in the airtight storage. The airtight storage may have led to a reduction in crude protein content depending on the temperature of the storage environment. High temperatures cause alterations in the chemical constituents of grains, such as lipids, carbohydrates, and proteins^18^. The acid value of the corn decreased with the increase in the storage time for all treatments, mainly for the batches of mixed corn (normal). Thus, it can be assumed that the storage effects were positive in maintaining the quality of maize and agree with the results of ashes. Reductions in the levels of ash over time were observed, regardless of the storage and type of corn grain.

The storage with aeration retained the initial characteristics, such as the ash content. At the end of storage, larger effects in broken grains were observed, with smaller percentages of ash. The final ash values were similar for all storage forms. The metabolic activity of the microorganisms associated grains and consumed organic matter metabolizing it to CO_2_, water, and other products, with heat release, which can become a structurally mineral composition without altering their total content being accelerated in deterioration cereals with moisture levels above 13–14%. Thus, the determined ash content is proportionately larger as the organic matter is consumed^35^. This study shows that the water content throughout the storage is low, which indicates a low deterioration of the grains, and thus a low ash content during the storage.

### Effects of drying and rehumidifying corn kernels during storage on the physical quality

**Figure 2:**
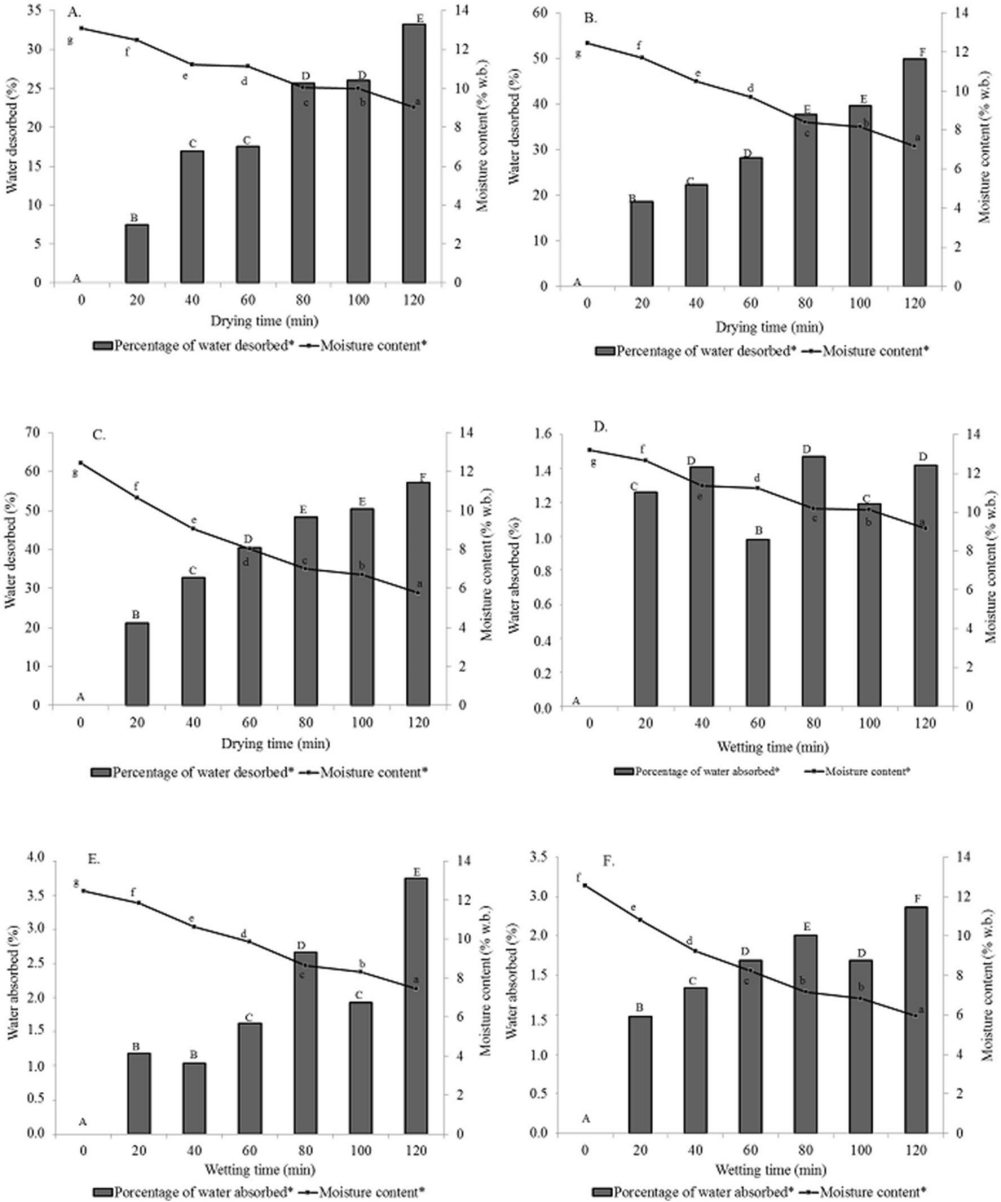
Variations in moisture content—desorbed and absorbed water during the drying and wetting procedures: (**A**,**D**) 80, (**B**,**E**) 100, and (**C**,**F**) 120 °C. *Significant at a probability of 5% in the Tukey test.

**Figure 3:**
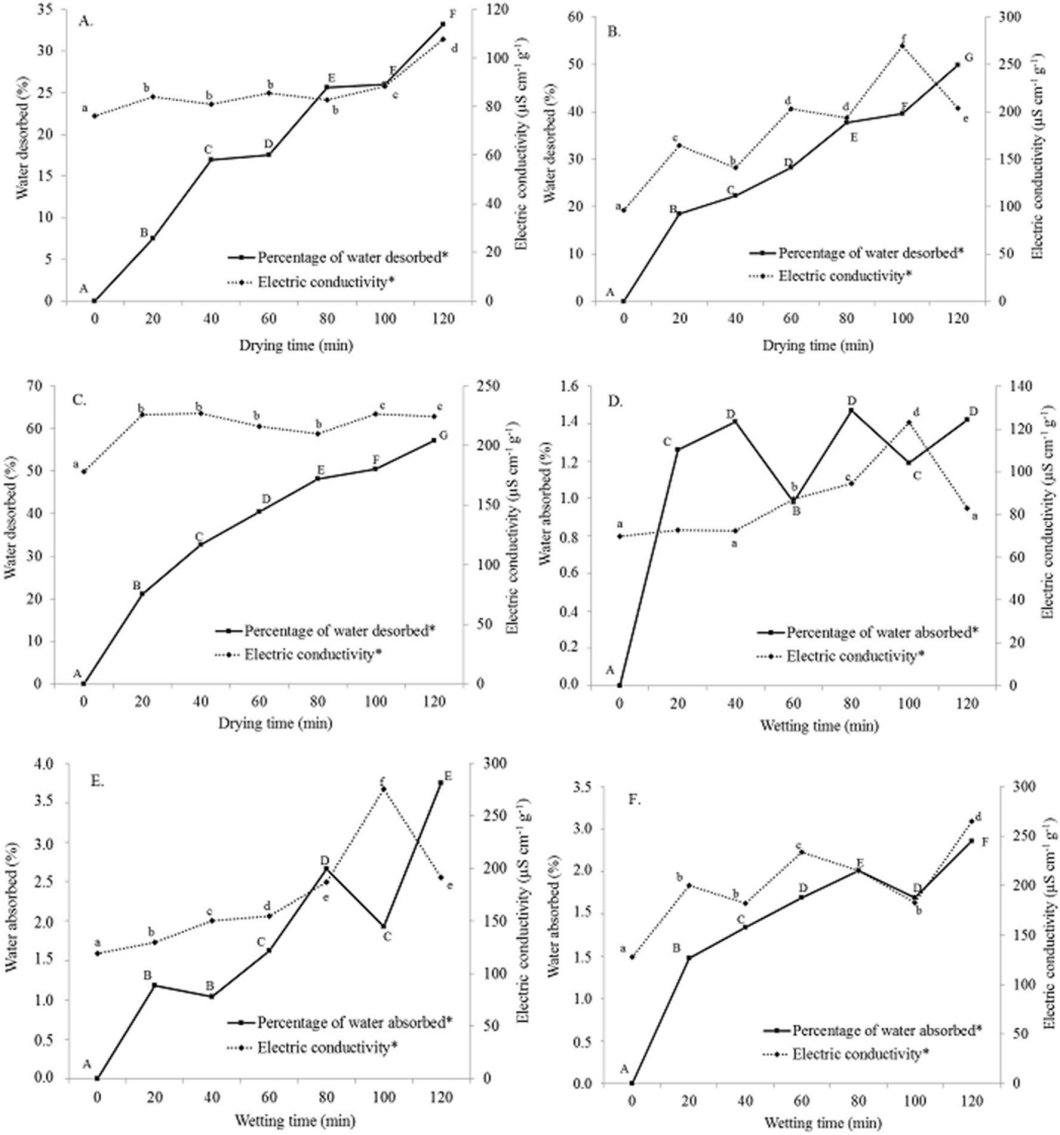
Variations in the percentage of dissolved and adsorbed water—electrical conductivity during the drying and moistening processes: (**A**,**D**) 80, (**B**,**E**) 100, and (**C**,**F**) 120 °C. *Significant at a probability of 5% in the Tukey test.

**Figure 4:**
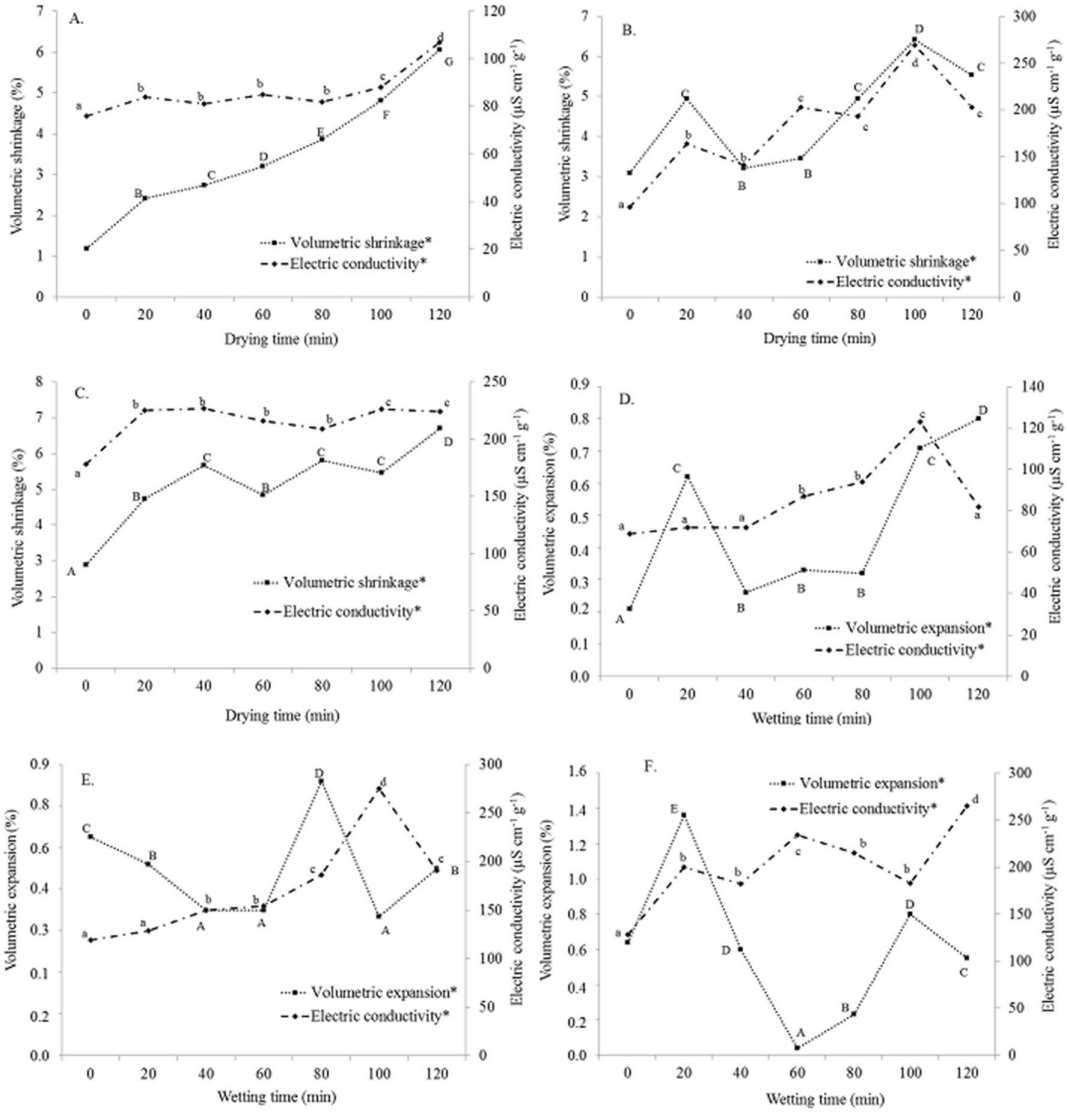
Volumetric shrinkage variation and expansion—electrical conductivity during the drying and wetting procedures: (**A**,**D**) 80, (**B**,**E**) 100, and (**C**,**F**) 120 °C. *Significant at a probability of 5% in the Tukey test.

Figures 2, 3 and 4 show that the physical processes of water desorption during the drying and rewetting with water absorption in the storage caused qualitative losses evaluated by the electrical conductivity test, shrinkage, and increase in the volume of corn kernels with different intensities, mainly depending on the time and temperature applied in the drying^21,55,56^.

The wetting of the grains with migration of moisture during the storage period is reflected in the quality of the grains, according to the results of the electrical conductivity tests. The intensity and number of cycles when the grain mass desorbed and absorbed water during the drying and remoistening processes during the storage aggravated the physical damage observed through cracks and ruptures of the cellular tissues constituting the grains. These results demonstrate that it is necessary to monitor and control the grain preprocessing operations so that the processes are homogeneous and safe^57–59^.

As shown in Table 5, the drying carried out at an air temperature of 120 °C provided better results regarding the acidity index. This contradicts the physical analyses presented above, although it can be justified. The drying at lower temperatures requires a longer period to reduce the water content of the grains. This leads to heating of the grain mass, associated with the high water content, which may cause grain fermentation and increase the acidity^60,61^. In the storage at room and cooling temperatures, a decrease in the acidity index after six months of evaluation was observed, regardless of the drying temperature. The results of this study are in agreement with previous studies where in undamaged grains stored at room temperature and moisture below 12%, small variations in acidity levels occurred^26,62^.

**Table 5:** Average results of 0.1 N NaOH acidity index (mL), crude protein (%), and ash (%) of corn kernels as a function of drying air temperature, condition and storage time.

Evaluation	Temperature air drying (°C)		Zero		Six months	
			Cooling (10 °C)	Ambient (23 °C)	Cooling (10 °C)	Ambient (23 °C)
Acidity level	80		2.46 Aa	2.46 Aa	1.47 Ba	2.05 Aa
	100		2.33 Aa	2.33 Aa	1.42 Ba	1.86 Aa
	120		1.94 Ab	1.94 Ab	1.31 Ba	1.98 Aa
Crude protein	80		8.72 Aa	8.72 Aa	8.09 Aa	7.82 Aa
	100		9.00 Aa	9.00 Aa	7.29 Ab	8.06 Aa
	120		8.00 Ab	8.00 Ab	7.67 Aa	7.82 Aa
Ashes	80		1.69 Aa	1.28 Bb	1.31 Aa	1.31 Aa
	100		1.65 Aa 1.53 Aa	1.55 Aa	1.06 Ab	1.06 Ab
	120			1.51 Aa	1.13 Ab	1.13 Ab

Averages followed by the capital letter on the line, for each storage time and lower case letters in the columns for each drying air temperature, do not differ, at 5% probability.

Table 5 shows that the increase in the drying air temperature reduced the percentage of crude protein in the grains. The same behavior was observed for the storage time, regardless of the temperature. Table 5 shows significant differences as a result of the storage time, rather than between the drying temperatures. All treatments reduced the percentage of crude protein during the storage, owing to the intrinsic chemical characteristics of the degradation and/or requirements of its constituents, in view of the physicochemical and biological factors of the storage conditions^17^. Table 5 shows that the increase in the drying temperature increased the ash percentage. The same behavior was observed with the increase in the storage time, regardless of the temperature. These results are consistent with previous studies on the percentages of ash or mineral constituents with significant differences between the drying temperatures and between the storage periods^7^.

### Multivariate analysis of main components and Pearson correlations for different drying and storage treatments for corn kernels

**Figure 5:**
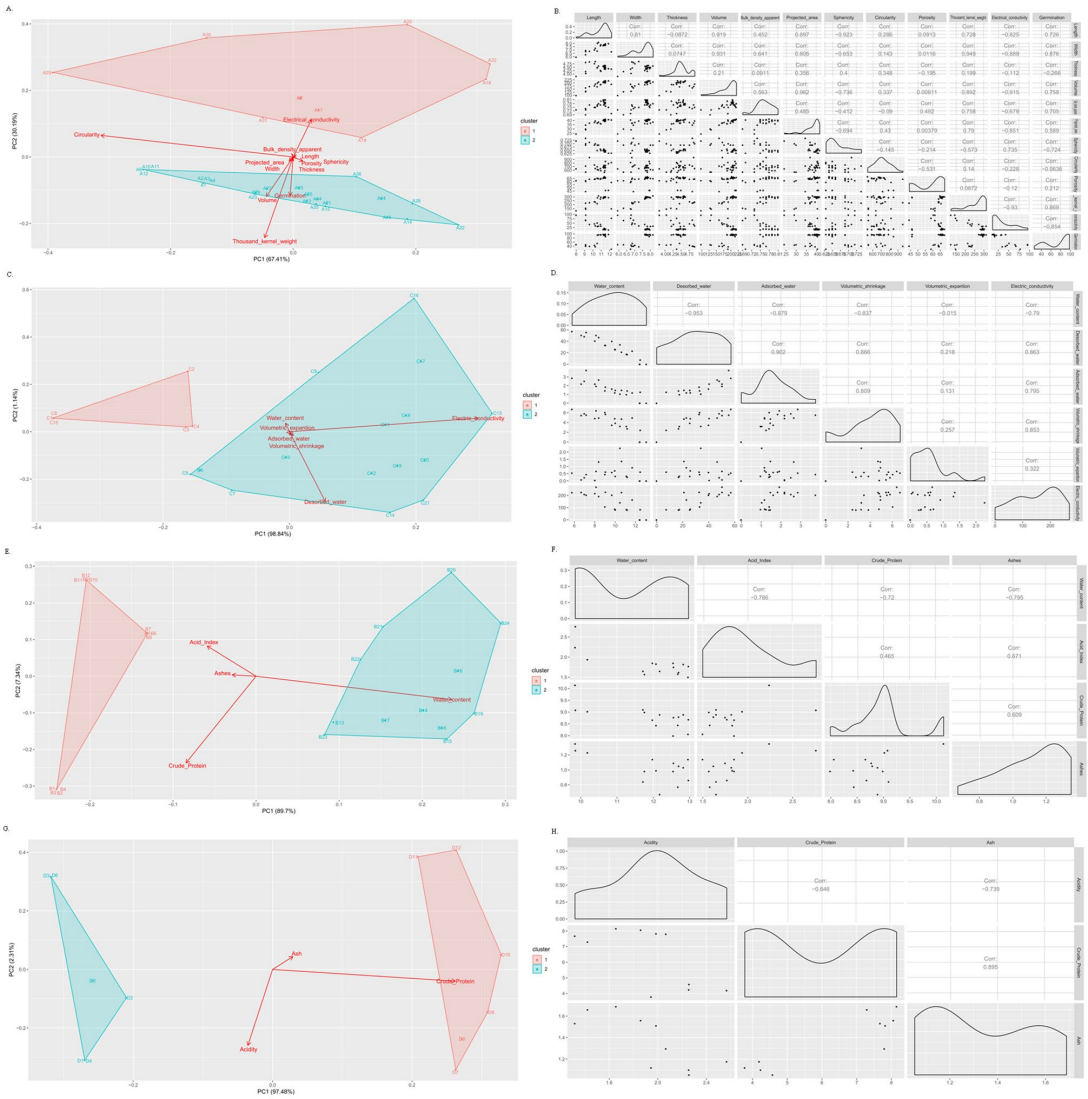
Principal component analysis (**A** physical quality, **E** physicochemical quality) and Pearson’s correlations (**B** physical quality, **F** physicochemical quality) of the effects of the whole, broken, and normal corn grain lots stored for different times under different storage conditions in hermetic systems, aerated and non-aerated metal silos, and bags on the physical quality. (**C**) Principal component analyses and (**D**) Pearson’s correlations of the effects of the initial water content of the grains and temperature of the drying air on the variations in water desorption and adsorption as a function of storage time and physical quality of the corn grain lots. (**G**) Principal component analyses and (**H**) Pearson’s correlations of the effects of the drying air temperature, environment, and storage time on the physicochemical quality of the corn grains.

The principal component analysis enables a simultaneous analysis of all variables evaluated in each experiment, in addition to forming homogeneous groups between the evaluated treatments. However, for an accurate evaluation of the biplot generated with the first two main components, it is desirable that the accumulated variance with their sum is larger than 80%. The accumulated variance in the first two main components was 97.60%, 99.98%, 97.04%, and 99.79% for the first, second, third, and fourth experiments, respectively (Fig. 5).

According to the principal component analysis of the effects of the whole, broken, and normal corn grain lots stored for different times under different storage conditions in hermetic systems, aerated and non-aerated metal silos, and bags on the physical quality (Fig. 5A), group 1 consisted of the smallest number of treatments (A5, A6, A7, A8, A17, A18, A19, A20, A29, A30, A31, and A32), which were associated with higher electrical conductivities. The grouping pattern of these treatments is mainly associated with the type of corn (broken corn).

The Pearson’s correlations for the effects of the whole, broken, and normal corn grain lots stored for different times under different storage conditions in hermetic systems, aerated and non-aerated metal silos, and bags on the physical quality were negative with most of the evaluated variables (Fig. 5B), except for the positive correlation with the sphericity. Group 2 consisted of the other treatments with emphasis on the variables germination, volume, and weight of one thousand grains. These variables had strong positive correlations.

According to the principal component analysis of the effects of the initial water content of the grains and temperature of the drying air on the variations in water desorption and adsorption as a function of the storage time and physical quality of corn grain lots, group 1 consisted of treatments C1, C2, C3, C4, C8, and C15 (Fig. 5C). These treatments had the highest water contents and lowest averages of the other evaluated variables. Group 2 consisted of the other treatments, with emphasis on the variables of volumetric expansion, adsorbed water, and volumetric shrinkage, which were strongly positively correlated with each other (Fig. 5D). These variables were strongly negatively correlated with the water content.

According to the principal component analysis of the effects of the whole, broken, and normal corn grain lots stored for different times under different storage conditions in hermetic systems, aerated and non-aerated metal silos, and bags on the physicochemical quality (Fig. 5E), group 1 consisted of the treatments with the highest averages of the variables acidity index, ash content, and crude protein content (B1, B2, B3, B4, B5, B6, B7, B8, B9, B10, B1, and B12). These variables were positively correlated with each other (Fig. 5F). The other treatments were allocated to group 2, with the highest water content. This variable was negatively correlated with the other evaluated variables. Notably, the clustering pattern obtained by the principal component analysis is associated with the storage time. The treatments in group 1 were associated with zero time, while those in group 2 with six months.

The results of the principal component analysis of the effects of the drying air temperature, environment, and storage time on the physicochemical quality of corn grains (Fig. 5G) were similar to those in Fig. 5E. The storage time was the main influencing factor. The treatments associated with zero storage time were in group 1 (Fig. 5G) with a higher acidity. This variable was negatively correlated with the other evaluated variables (crude protein and ash contents, Fig. 5H). Group 2 consisted of the individuals associated with the storage period of six months, with higher crude protein and ash contents, which were positively correlated variables.

### Final considerations

The association of corn drying and storage conditions enabled to define the best strategy for the preservation of grains in the postharvest on the field scale of production. The increase in the drying air temperature accelerated the reduction in the water content of corn until the storage condition was met. The storage time of six months influenced the physical properties and reduced the physicochemical quality of corn in the storage at 23 °C. However, the storage at 10 °C maintained the quality of the physical and physicochemical properties of the corn grains over six months. The alternatives of storage with and without aeration in bags and airtight environments did not influence the physical properties of corn kernels. However, the hermetic and aerated storage systems maintained the chemical quality over the storage period. The different storage conditions, with and without aeration, in bags and in the airtight environment, did not maintain the quality of the stored grains with defects and broken kernels. The presence of deteriorated grains had a larger influence on the final quality of the corn lots. The increase in water content due to the wetting during the storage period caused losses in the quality of the corn kernels, similar to the drying for the conditions of safe storage water contents. The process control with homogenization of healthy and whole grain corn lots subjected to drying with an air temperature of 80 °C and storage in silos with natural aeration yielded satisfactory results, which were equivalent to those of uncontrolled drying and storage under airtight conditions and at low temperatures.

## Materials and methods

### Characteristics of the experiment

**Figure 6:**
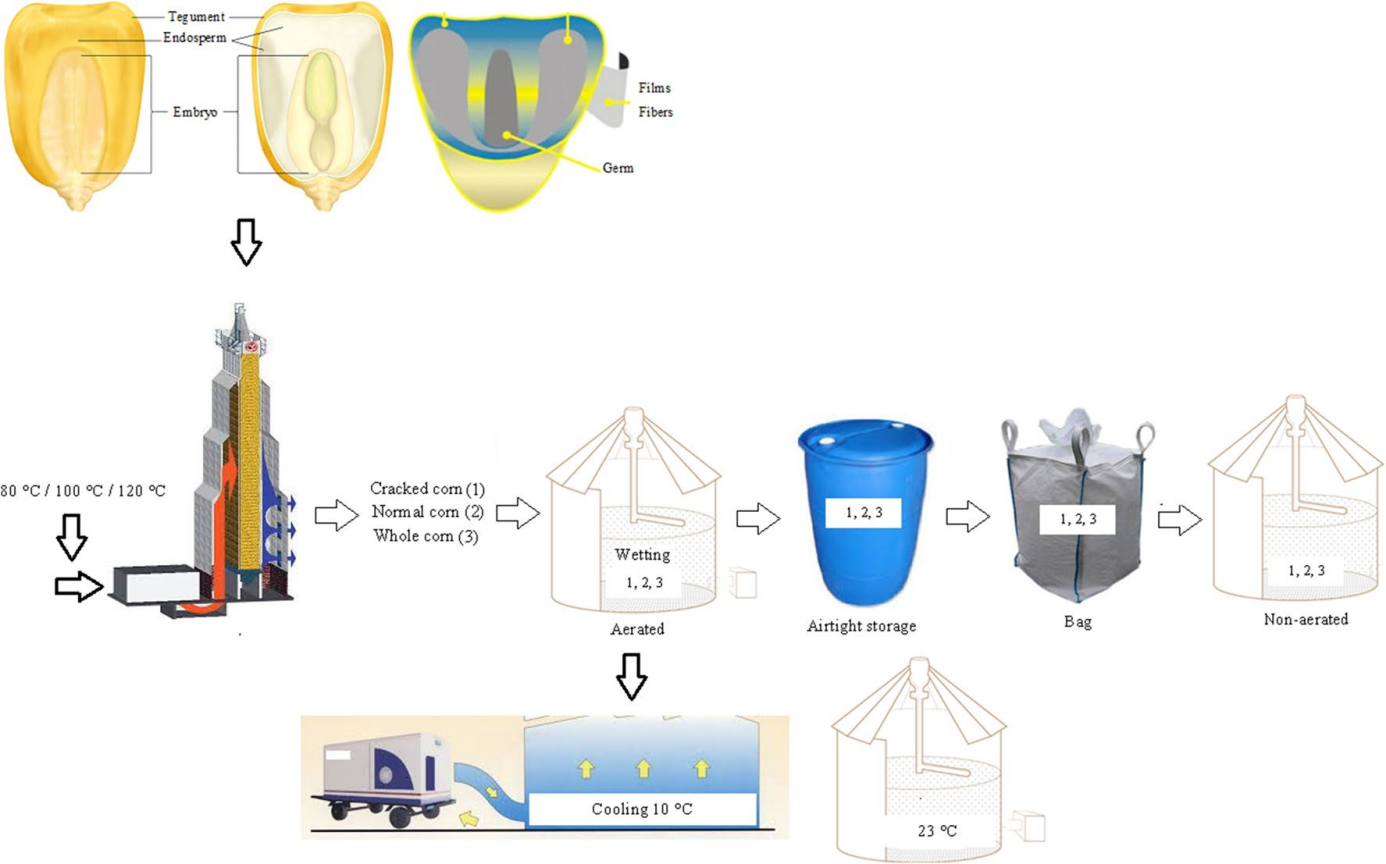
Material flow for the field experiment.

The used maize was classified as hard-type transgenic hybrid corn kernels *Herculex 30S31H*. The corn kernels were harvested manually on the cob, at random, with water contents of ~ 27%. Subsequently, the samples were sent to a manual track, and then subjected to cleaning to remove impurities. The samples were then subjected to drying and storage under different conditions (Fig. 6).

### Evaluation of the corn in the drying and storage processes on the field scale

Corn kernels were subjected to drying in a commercial continuous-flow dryer on a full scale (KW Dryer, capacity: 100 ton h^−1^, air flow: 220 m^3^ h^−1^) in three separate tests, with air temperatures of 80, 100, and 120 °C. The dried corn lots were then stored for six months in four storage systems, the hermetic environment, bags, aerated vertical silo, and non-aerated vertical silo. In the hermetic environment, 100-L polyethylene terephthalate containers were used, while for the storage of grains in bags, permeable nylon packages with a capacity of 1000 kg were used. For the storage of grains with and without aeration, vertical silos with a capacity of 20 ton were used. For each storage system, three types of corn grain lots were used, healthy grains, completely clean and without defects (clean grains), grains with 2–4% impurities (normal grains), and grain lots with 5–7% broken grains (broken grains). Three samples of each lot were collected in the upper, lower, and middle parts of the airtight container and in the upper and lower parts of the vertical silos, at times of zero, three, and six months for the evaluation of the physical quality and at times of zero and six months for the assessment of the physicochemical quality.

### Physical quality of the maize grains subjected to drying reprocesses after rewetting during storage

Traditionally, during the storage of corn lots, moisture migration processes are observed in the grain mass according to the environmental conditions established for a hygroscopic balance, which can influence the initial quality. To understand the effects of the moisture migration on the grain mass and loss of quality of the grains stored using the different technologies and forms of storage presented in “Quality of corn grains subjected to drying and storage under different production-scale conditions” section, an experimental study was carried out with dry corn grains at temperatures of 80, 100, and 120 °C according to the description in “Quality of corn grains subjected to drying and storage under different production-scale conditions” section. The grains were then randomly sampled. One hundred and fifty grains were collected from each sample to be stored in controlled environments (bio-oxygen demand (BOD) chamber) at 10 °C and relative air humidity of 90%, simulating an intensive wetting for 0, 20, 40, 60, 80, 100, and 120 min. The grains were then dried at the same air temperature during the same wetting period. For each case, the water content, amounts of absorbed and desorbed water, kernel volumes of contraction and expansion, and electrical conductivity were measured to evaluate the deterioration of cellular tissues.

### Quality of the corn kernels subjected to drying and stored at low temperatures

To demonstrate the effects of the storage technologies (“Quality of corn grains subjected to drying and storage under different production-scale conditions” section) and changes due to the migration of moisture in the grain mass according to the experiment in “Effects of drying and rehumidifying corn kernels during storage on the physical quality” section, a sample was collected under drying conditions at 80, 100, and 120 °C for storage in two controlled environments, refrigeration at 10 °C and relative humidity of 40% and at 23 °C and relative humidity of 60% over six months. The environment and status of the grains were monitored to characterize the physicochemical qualities at the beginning and end of storage.

### Physical and physicochemical quality analyses of the corn grains

The water content was determined by the standard oven method at 105 ± 1 °C for 24 h with three replications^63^. The physical parameters of length, width, thickness, volume, projected area, sphericity, circularity, porosity, apparent bulk density, and thousand kernel weight were determined^64^. Electrical conductivity^65^, crude protein, acidity, and ash analyses were also performed^66^.

### Statistical analysis

The physical and physicochemical quality data were evaluated by an analysis of variance, Tukey’s test at probabilities of 1% and 5%, and linear regression. To verify the interrelationship between the variables and treatments of each experiment, the data were used for a principal component analysis. A biplot was produced with the first two main components owing to the ease of interpretation of these results. In the biplot, two clusters were defined to use the *k*-means algorithm, which groups treatments whose centroids are closest until no significant variation in the minimum distance of each observation to each centroid occurs. In addition, a Pearson correlation graph was generated for each experiment. These analyses were performed with the aid of the “ggfortify” package of the free application R^67^ (Table 6).

**Table 6:** Experimental parameters to determine the quality of maize according to different drying air temperatures storage systems and durations.

First evaluation			
Storage times (months)	Corn type	Storage type	Groupings
0	Whole Corn	Airtight	A1
0	Whole Corn	Bag	A2
0	Whole Corn	Non-aerated	A3
0	Whole Corn	Aerated	A4
0	Broken Corn	Airtight	A5
0	Broken Corn	Bag	A6
0	Broken Corn	Non-aerated	A7
0	Broken Corn	Aerated	A8
0	Normal Corn	Airtight	A9
0	Normal Corn	Bag	A10
0	Normal Corn	Non-aerated	A11
0	Normal Corn	Aerated	A12
3	Whole Corn	Airtight	A13
3	Whole Corn	Bag	A14
3	Whole Corn	Non-aerated	A15
3	Whole Corn	Aerated	A16
3	Broken Corn	Airtight	A17
3	Broken Corn	Bag	A18
3	Broken Corn	Non-aerated	A19
3	Broken Corn	Aerated	A20
3	Normal Corn	Airtight	A21
3	Normal Corn	Bag	A22
3	Normal Corn	Non-aerated	A23
3	Normal Corn	Aerated	A24
6	Whole Corn	Airtight	A25
6	Whole Corn	Bag	A26
6	Whole Corn	Non-aerated	A27
6	Whole Corn	Aerated	A28
6	Broken Corn	Airtight	A29
6	Broken Corn	Bag	A30
6	Broken Corn	Non-aerated	A31
6	Broken Corn	Aerated	A32
6	Normal Corn	Airtight	A33
6	Normal Corn	Bag	A34
6	Normal Corn	Non-aerated	A35
6	Normal Corn	Aerated	A36

Second evaluation			
Drying air temperature (°C)	Time (min)	Water content (w.b.) (%)	Groupings
80	0	13,08	C1
80	20	12,49	C2
80	40	11,21	C3
80	60	11,13	C4
80	80	10,04	C5
80	100	9,99	C6
80	120	9,02	C7
100	0	12,45	C8
100	20	11,70	C9
100	40	10,50	C10
100	60	9,70	C11
100	80	8,41	C12
100	100	8,15	C13
100	120	7,18	C14
120	0	12,42	C15
120	20	10,65	C16
120	40	9,07	C17
120	60	8,03	C18
120	80	6,99	C19
120	100	6,69	C20
120	120	5,78	C21

Third assessment			
Storage Times (months)	Corn type	Storage Type	Groupings
0	Whole Corn	Airtight	B1
0	Whole Corn	Bag	B2
0	Whole Corn	Non-aerated	B3
0	Whole Corn	Aerated	B4
0	Broken Corn	Airtight	B5
0	Broken Corn	Bag	B6
0	Broken Corn	Non-aerated	B7
0	Broken Corn	Aerated	B8
0	Normal Corn	Airtight	B9
0	Normal Corn	Bag	B10
0	Normal Corn	Non-aerated	B11
0	Normal Corn	Aerated	B12
6	Whole Corn	Airtight	B13
6	Whole Corn	Bag	B14
6	Whole Corn	Non-aerated	B15
6	Whole Corn	Aerated	B16
6	Broken Corn	Airtight	B17
6	Broken Corn	Bag	B18
6	Broken Corn	Non-aerated	B19
6	Broken Corn	Aerated	B20
6	Normal Corn	Airtight	B21
6	Normal Corn	Bag	B22
6	Normal Corn	Non-aerated	B23
6	Normal Corn	Aerated	B24

Fourth evaluation			
Storage times (months)	Storage conditions (°C)	Drying air temperature (°C)	Groupings
0	23	80	D1
0	23	100	D2
0	23	120	D3
0	10	80	D4
0	10	100	D5
0	10	120	D6
6	23	80	D7
6	23	100	D8
6	23	120	D9
6	10	80	D10
6	10	100	D11
6	10	120	D12
